# Supplementary material for: NMR-Based Metabolomics Reveals Effects of Water Stress in the Primary and Specialized Metabolisms of Bauhinia ungulata L. (Fabaceae)
Source: Metabolites. 2023 Mar 3;13(3):381. doi: 10.3390/metabo13030381 (PMC10053921; doi:10.3390/metabo13030381)
Supplement: Supplementary file 1 [file metabolites-13-00381-s001.zip › metabolites-2227494-supplementary.pdf]

Supporting information

NMR-Based Metabolomics Reveals Effects of Water Stress in the Primary and Specialized Metabolisms of *Bauhinia ungulata* L. (Fabaceae)

Ana Júlia Borim de Souza [1], Fernanda Maria Marins Ocampos [2,*], Rafael Catoia Pulgrossi [3], Anne Lígia Dokkedal [1], Luiz Alberto Colnago [2], Inês Cechin [1] and Luiz Leonardo Saldanha [1,*]

[1] Faculty of Sciences, São Paulo State University (UNESP), Bauru 17033-360, SP, Brazil
[2] Embrapa Instrumentation, São Carlos 13560-970, SP, Brazil
[3] Department of Statistics, Federal University of São Carlos (UFSCar), São Carlos 13565-905, SP, Brazil
* Correspondence: fmmocampos@gmail.com (F.M.M.O.); lluizsaldanha@gmail.com (L.L.S.)

Correspondence:

Luiz Leonardo Saldanha Faculty of Sciences, São Paulo State University (UNESP), CEP 17033-360, Bauru, São Paulo, Brazil.
Email: lluizsaldanha@gmail.com

Fernanda Maria Marins Ocampos Brazilian Agricultural Research Corporation (Embrapa), CEP 13560-970, São Carlos, São Paulo, Brazil.
Email: fmmocampos@gmail.com

Table S1. ^{1}H NMR (600 MHz) and ^{13}C NMR data (150 MHz) of amino acids identified in the hydroalcoholic leaves extract of *Bauhinia ungulata* (CD_3OD:D_2O - 7:3 v/v).

	**19**			**20**			**21**			**22**		
**Position**	**δH, mult. (*J* Hz)**	**δC**	**HMBC**	**δH, mult. (*J* Hz)**	**δC**	**HMBC**	**δH, mult. (*J* Hz)**	**δC**	**HMBC**	**δH, mult. (*J* Hz)**	**δC**	**HMBC**
**1**		174.9			n.d.			172.5			173.5	
**2**	3.57, m	50.7	n.d.	n.d.	60.5		2.67, dd (16.9; 8.5)<br>2.84, dd (16.9; 3.7)	34.4 CH2	1, 3/ 1, 3, 4	3.93, m	62.3	1, 3, 4, 5
**3**	1,37, d (7.2)	16.2	3, 1	n.d.	29.4			51.5		1.98, m/ 2.22, m	29.2	1, 2, 4, 5
**4**				0.94, d (7.0)/ 0.89 d (7.0)	18.2/ 16.5	2, 3, 4		174.9		1.89, m	23.9	1, 3, 5
**5**										3.19, m/ 3.29, m	46.1	1, 3, 4, 5

*n.d: Not detected by the HSQC

Table S2. ^{1}H NMR (600 MHz) and ^{13}C NMR data (150 MHz) of polyols identified in the hydroalcoholic leaves extract of *Bauhinia ungulata* (CD_3OD:D_2O - 7:3 v/v).

	**23**			**24**			**25**			**26**		
**Position**	**δH, mult. (*J* Hz)**	**δC**	**HMBC**	**δH, mult. (*J* Hz)**	**δC**	**HMBC**	**δH, mult. (*J* Hz)**	**δC**	**HMBC**	**δH, mult. (*J* Hz)**	**δC**	**HMBC**
**1**	5.05, d (3.7)	92.9	3	4.44, d (7.9)	96.5	n.d	3.83, q (5.9; 1.8)	72.0	1, 2, 3, 4, 6	5.29, d (3.8)	92.3	2, 6, 2'
**2**	n.d	n.d	n.d	n.d	n.d	n.d	3.66, dd (9.8; 2.6)	70.5	1, 3	n.d	72.9	n.d
**3**	n.d	73.2	n.d	n.d	n.d	n.d	3.19, t (9.8)	83.2	1, 2, 6, OCH_3	n.d	n.d	n.d
**4**	n.d	n.d	n.d	n.d	n.d	n.d	3.50, t (9.8)	72.6	2, 3, 6	n.d	n.d	n.d
**5**	n.d	n.d	n.d	n.d	n.d	n.d	3.61, dd (9.8; 2.6)	71.1	1, 4, OCH_3	n.d	n.d	n.d
**6**	n.d	n.d	n.d	n.d	n.d	n.d	3.83, q (5.9; 1.8)	70.5	1, 2, 3, 4, 6	3.53, m	62.1	2'
**1'**										n.d	61.8	n.d
**2'**											103.9	1.
**3'**										4.04, d (8.4)	77.1	4', 5'
**4'**										3.91, m	74.2	2', 3', 5'
**5'**										3.71, m	81.9	2', 3', 4'
**6'**										n.d	n.d	n.d
**OCH_3**							3.49, s	59.7	3, 5			

*n.d: Not detected by the HSQC

Table S3. ^{1}H NMR (600 MHz) and ^{13}C NMR data (150 MHz) of phenolic compounds identified in the hydroalcoholic leaves extract of *Bauhinia ungulata* (CD_3OD:D_2O - 7:3 v/v).

**Position**		**27**			**28**			**29**			**30**			**31**	
	**δ_H, mult. (*J* Hz)**	**δ_C**	**HMBC**	**δ_H, mult. (*J* Hz)**	**δ_C**	**HMBC**	**δ_H, mult. (*J* Hz)**	**δ_C**	**HMBC**	**δ_H, mult. (*J* Hz)**	**δ_C**	**HMBC**	**δ_H, mult. (*J* Hz)**	**δ_C**	**HMBC**
**1**		128.9			121.6			n.d.							
**2**	7.00, d (8.2)	131.5	2, 3, 4, 5, 6, 7	6.88, s	108.8	1, 3, 4, 5, 6, 7	7.04, s	108.7	3, 4, 5, 6, 7		144.5			n.d.	
**3**	6.60, d (8.2)	114.6	1, 3, 4, 5		144.7			145.1			n.d.			n.d.	
**4**		154.8			.136.6			136.5			n.d.			n.d.	
**5**	6.60, d (8.2)	114.6	1, 3, 4, 5		144.7			145.1			161.9			161.9	
**6**	7.00, d (8.2)	131.5	2, 3, 4, 5, 6, 7	6.88, s	108.8	1, 2, 3, 4, 5, 7	7.04, s	108.7	2, 3, 4, 5, 7	6.19, d (2.2)	99.0	5, 7, 8, 10	6.19, d (2.2)	99.0	5, 7, 8, 10
**7**	2.88, m	39.7	1, 2, 6, 8		158.7			159.1			164.2			164.2	
**8**	2.94, m	39.7	7, 1’, 2’, 6’							6.40, d (2.2)	94.1	6, 7, 9, 10	6.38, d (2.2)	94.1	6, 7, 9, 10
**9**											157.4			157.4	
**10**											104.7			104.7	
**1’**		128,8									121.7			121.7	
**2’**	7.00, d (8.2)	131.5	8, 2’, 3’, 4’, 5’, 6’							7.68, d (8.8)	130.9	2’, 4’, 6’	7.26, d (2.2)	116.1	3’, 4’, 6’
**3’**	6.60, d (8.2)	114.6	1’, 3’, 4’, 5’							6.90, d (8.8)	115.6	2, 1’, 3’, 5’		144.9	
**4’**		154.8									159.4			148.2	
**5’**	6.60, d (8.2)	114.6	1’, 3’, 4’, 5’							6.90, d (8.8)	115.6	2, 1’, 3’, 5’	6.62, d (8.4)	115.3	1’, 3’
**6’**	7.00, d (8.2)	131.5	8, 2’, 3’, 4’, 5’, 6’							7.68, d (8.8)	130.9	2’, 4’, 6’	7.23, dd (8.4; 2.2)	122.0	2’, 4’

[n.d]: Not detected by the HSQC*

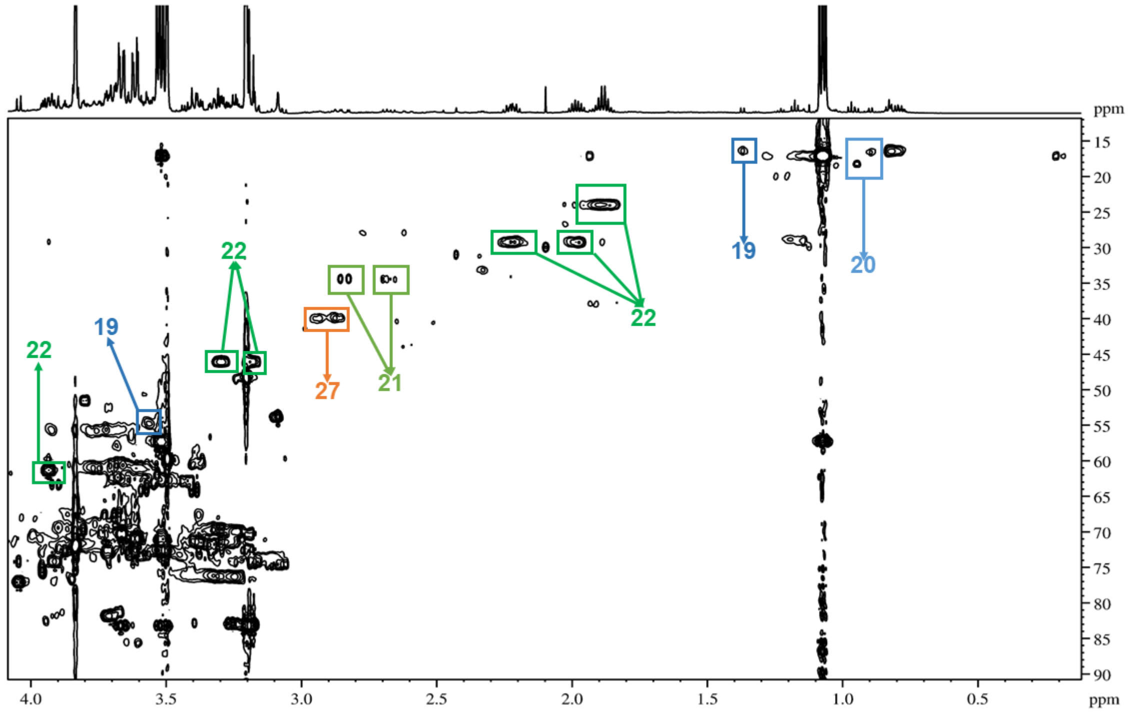

Figure S1. Region of the HSQC correlation map relative to the amino acids identified in *B. ungulata* extract ($CD_3OD:D_2O$ - 7:3 v/v).

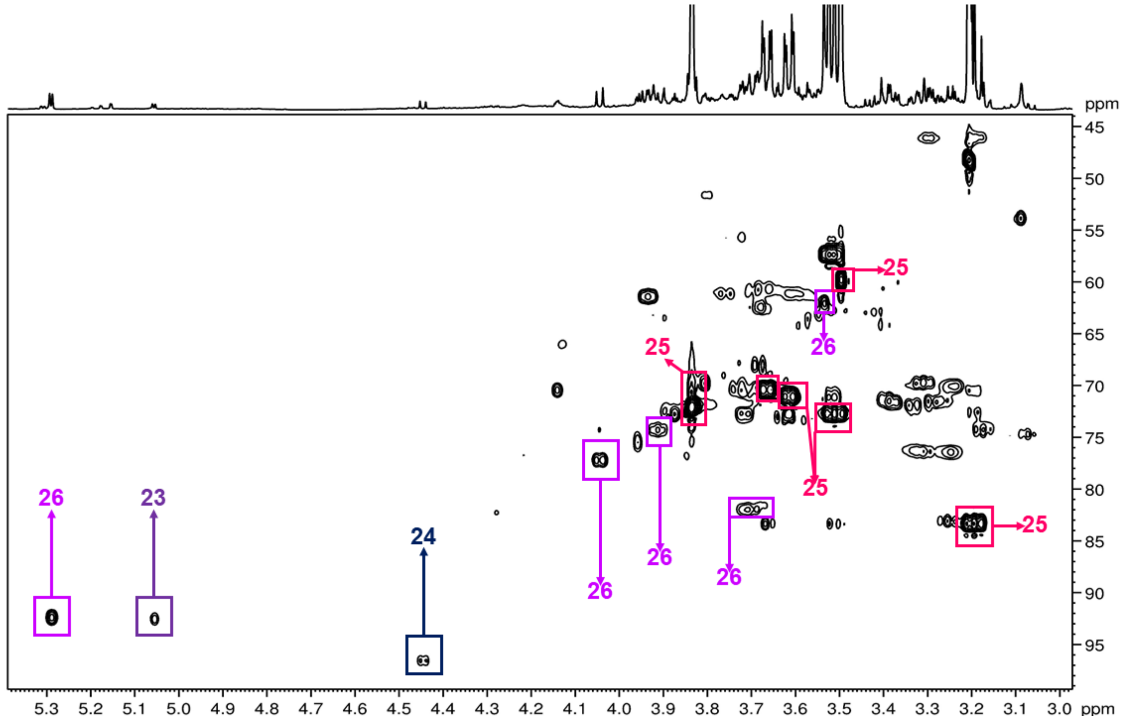

Figure S2. Region of the HSQC correlation map relative to the polyols identified in *B. ungulata* extract ($CD_3OD:D_2O$ - 7:3 v/v).

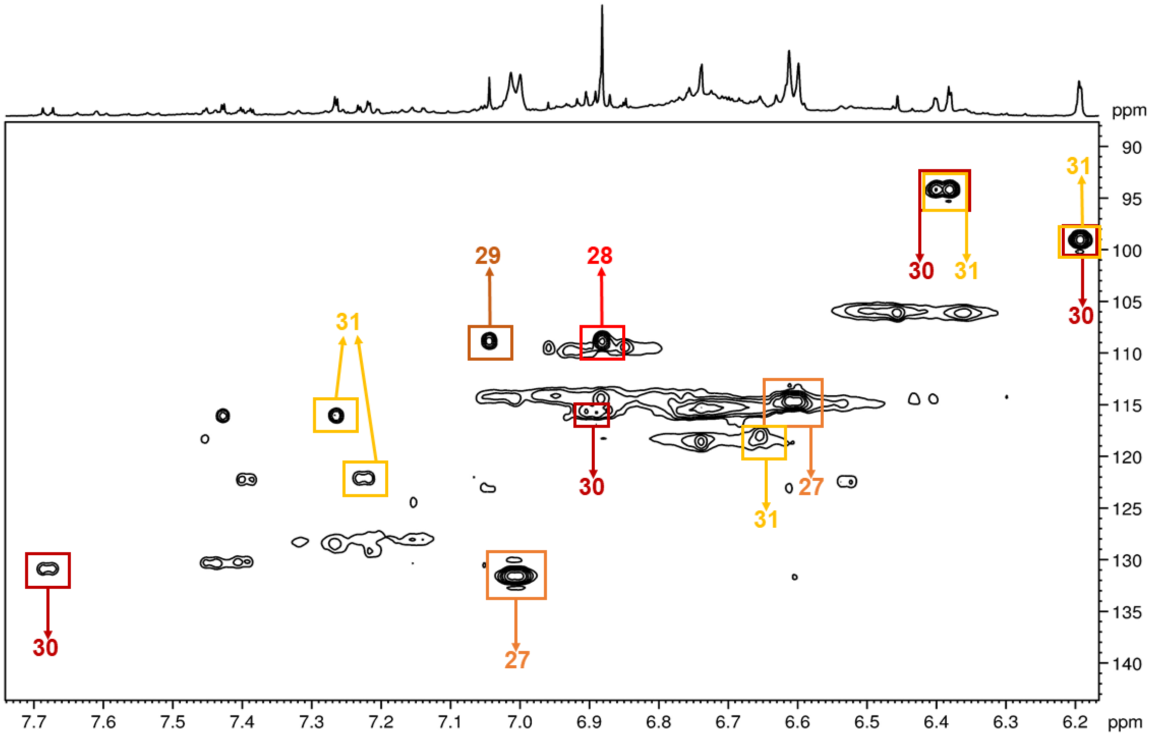

Figure S3. Region of the HSQC correlation map relative to the phenolics identified in *B. ungulata* extract (CD_3OD:D_2O - 7:3 v/v).

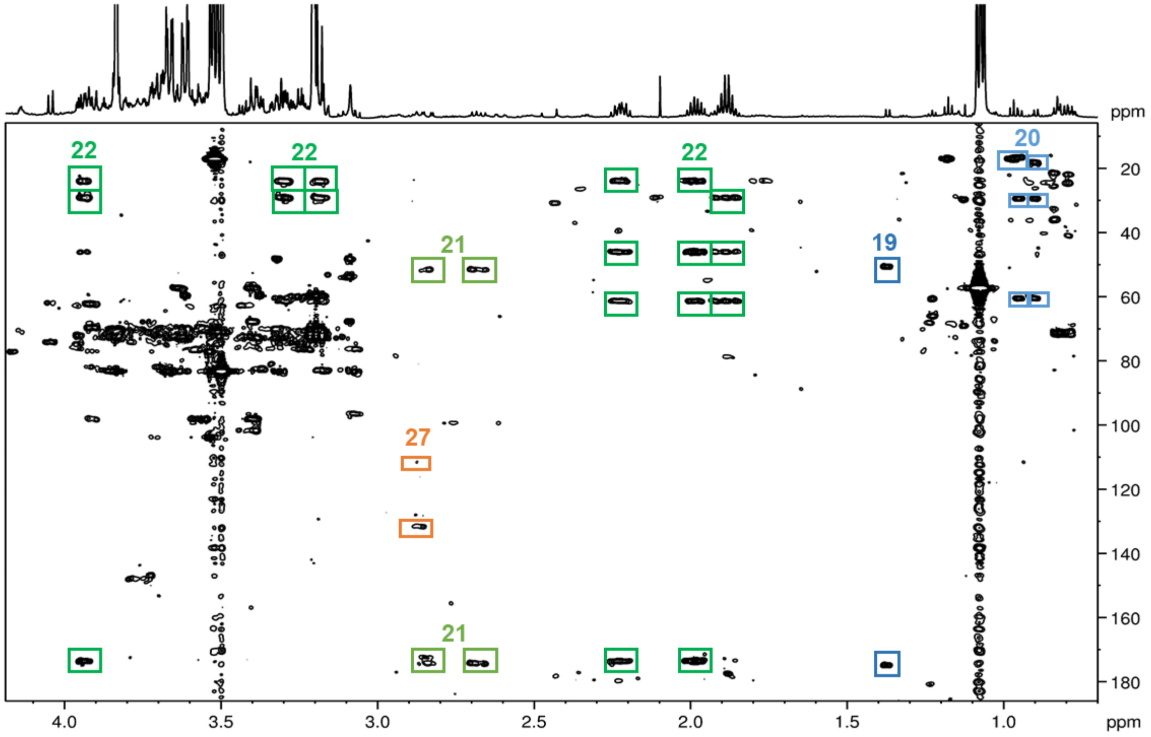

Figure S4. Region of the HMBC correlation map relative to the amino acids identified in *B. ungulata* extract (CD_3OD:D_2O - 7:3 v/v).

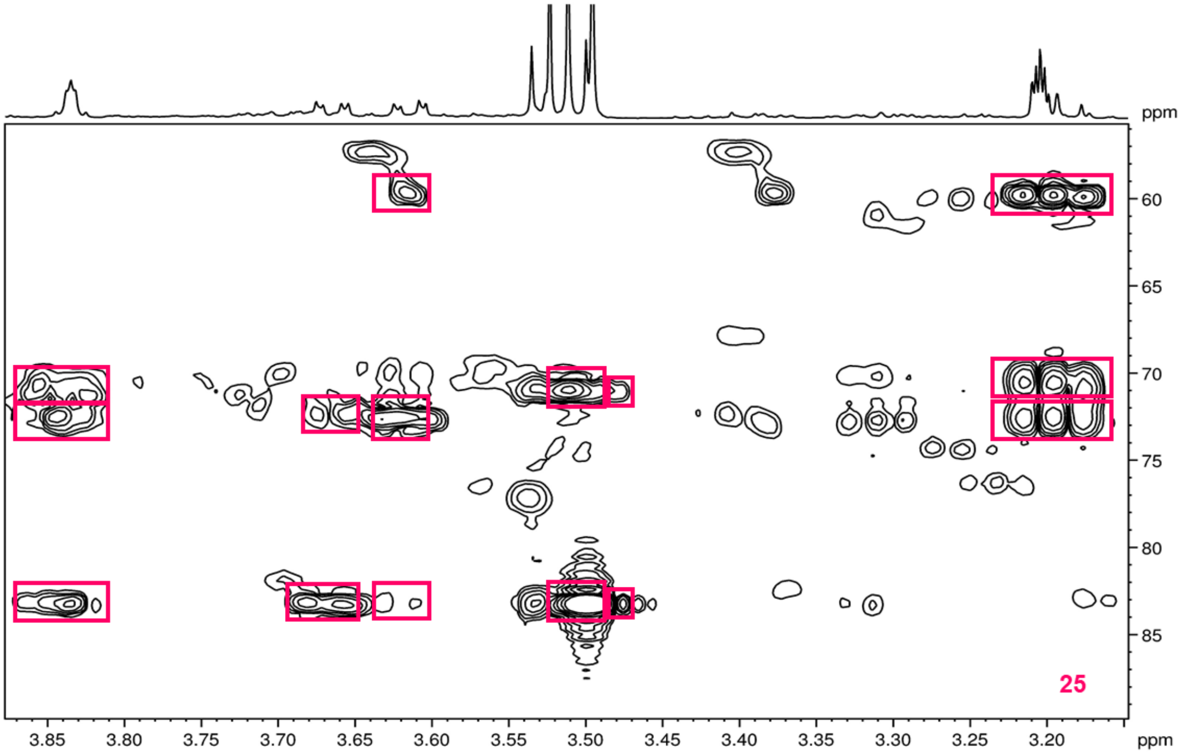

Figure S5. Region of the HMBC correlation map relative to the compound D-pinitol (25) identified in *B. ungulata* extract (CD_3OD:D_2O - 7:3 v/v).

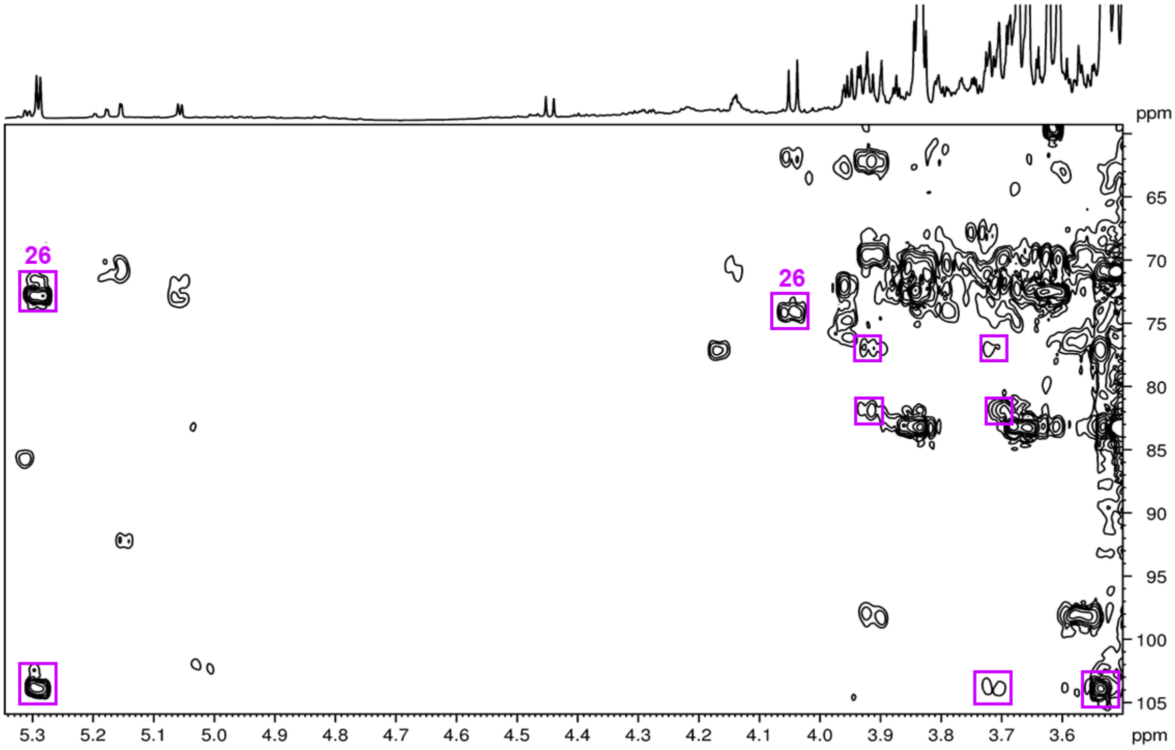

Figure S6. Region of the HMBC correlation map relative to the compound Sucrose (26) identified in *B. ungulata* extract (CD_3OD:D_2O - 7:3 v/v).

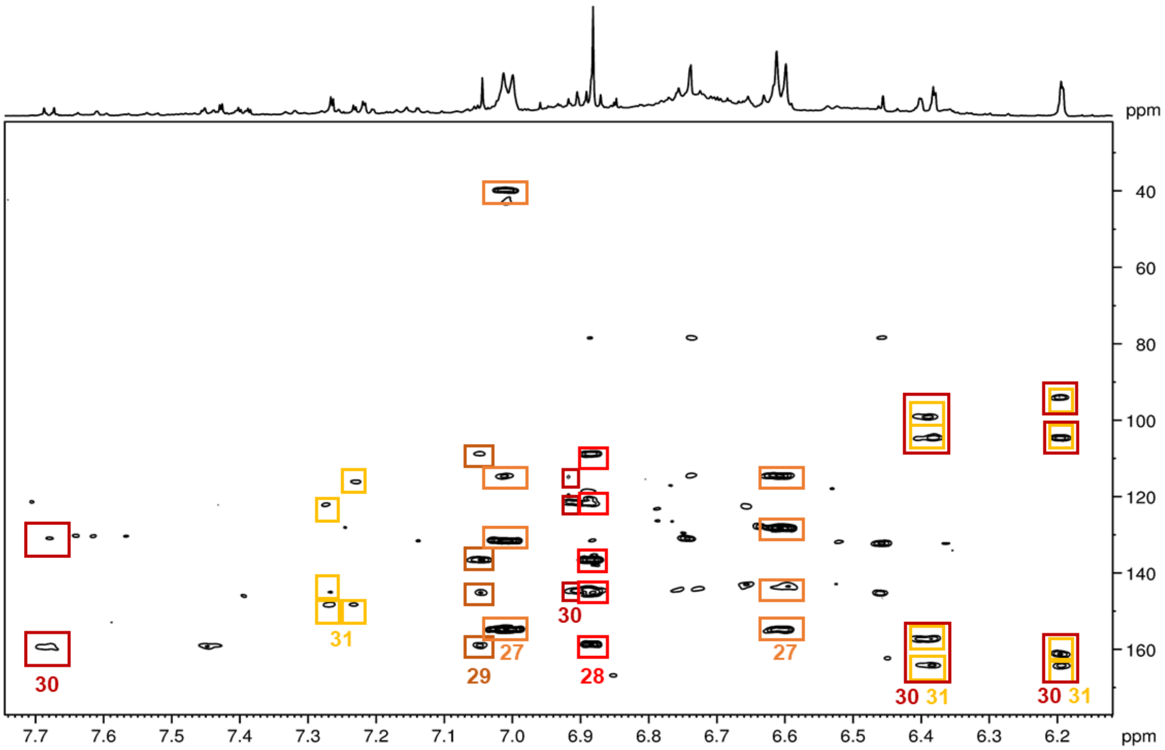

Figure S7. Region of the HMBC correlation map relative to the phenolics identified in *B. ungulata* extract ($CD_3OD:D_2O$ - 7:3 v/v).

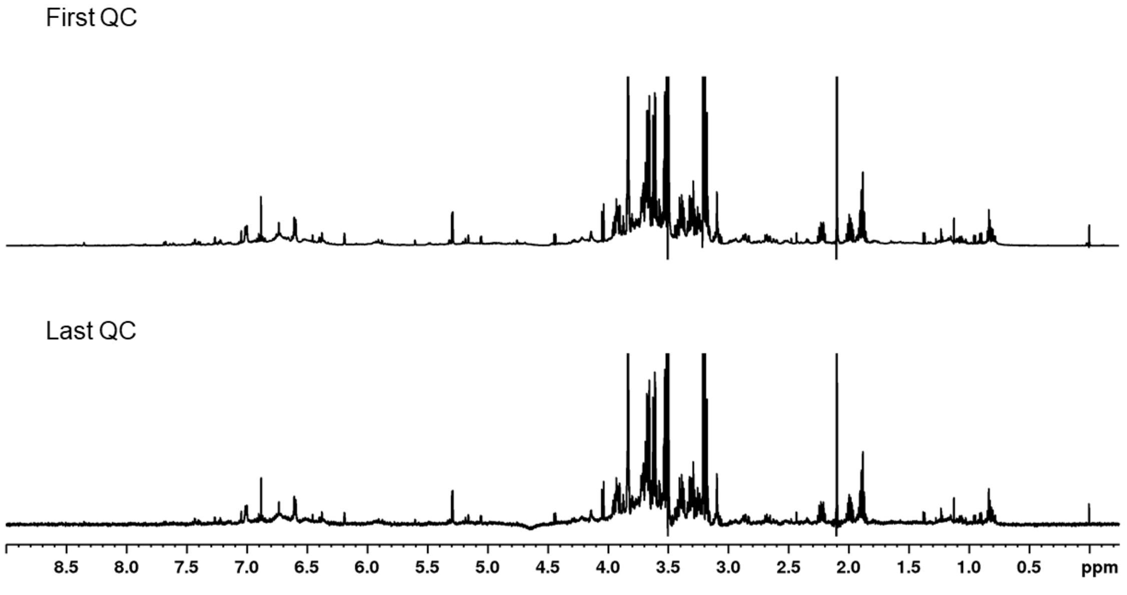

Figure S8. Quality Control (QC) sample validation of the first acquisition (upper part of the figure), and of the last acquisition, (lower part of the figure) of representative ^{1}H-NMR profile of the hydroalcoholic leaves extract of *Bauhinia ungulata* specimens.
